# 

**Published:** 1917-09-22

**Authors:** 


### CONTENTS.

| | PAGE |
|---|---|
| **The War Office and the Medical Profession...** | 499 |
| **Auxiliary Hospitals and their Uses ... ...** | 500 |
| **Hospital and Institutional News ... ... ...** | 501 |
| The Medical Certificate Again—Artificial Limbs and Research—The King of Spain and Hospital Ships—The Waiting List at Cardiff—Re-education of the Wounded—A Large Charitable Bequest—£200,000 for A Home of Rest—Moose Jaw Military Hospital—Poor-Law Infirmaries and Soldiers' Wives—A Petition to the Local Government Board—London Air-raid Refugees—The Food Controller's Manifesto—The Ship's Surgeon, 1865-1917—The Children of Blinded Soldiers—Home for Disabled Sailors and Soldiers at Newcastle—Personal Decoration by President Poincaré—A Useful Life Cut Short—The British Red Cross in Italy—A Classic Hospital Design—A Cottage Hospital for Castleford—Swimming Baths with Unchanged Water—The Future of Samaritan Funds—Refusal to Attend Chapel—The Deadlock at Merthyr—The Baby Problem—The Stuffy Class-room—Synthetic Alcohol—Sterilised Paper as Surgical Dressings—This Week's Drug Market. | |
| **German Genius and Methods ... ... ... ...** | 507 |
| Is their Reputation Justified by their Real Worth? | |
| **Hospitals on Emigrant Ships ... ... ...** | 508 |
| New Board of Trade Rules. | |
| **How the Problem of the Blind Can be Solved** | 509 |
| The Report of the Departmental Committee. | |
| **Medical Officers and the Supply of Drugs ...** | 510 |
| **The Catgut Ligature ... ... ... ... ...** | 511 |
| A Technique for its Use in Civil Practice. | |
| **Nursing Affairs in Ireland ... ... ... ...** | 512 |
| A State of Unrest. A New Movement on Foot. | |
| **The Matrons' and Sisters' Department ...** | 513 |
| Matrons in Council: VII. The Future of the Nursing Profession. | |
| Round the Hospitals. | |
| Nurses and Night Duty—The Effect of Living "Upside Down"—Two Possible Rearrangements—College of Nursing Meetings—The Private Nurse in India—A Neglected Food. | |
| **Communal Cookery ... ... ... ... ... ...** | 516 |
| A New Opportunity for Educated Women. | |
| **Nature Studies in Macedonia—II. ... ...** | 517 |
| **The Editor's Letter-Box ... ... ... ...** | iv |
| **The Institutional Worker ... ... (Suppl.)** | 1 |

